# Percutaneous Mechanical Pulmonary Thrombectomy in a Patient With Pulmonary Embolism as a First Presentation of COVID-19

**DOI:** 10.7759/cureus.9506

**Published:** 2020-08-01

**Authors:** Gilles J Hoilat, Ceren Durer, Seren Durer, Pratishtha Gupta

**Affiliations:** 1 Internal Medicine, State University of New York Upstate Medical University, Syracuse, USA

**Keywords:** covid-19, saddle pulmonary embolism, mechanical thrombectomy

## Abstract

There has been a high incidence of thromboembolic diseases in patients with coronavirus disease 2019 (COVID-19) pneumonia. We present a case of a healthy 32-year-old male with no past medical history who presented with shortness of breath, tested positive for COVID-19, and was found to have a large acute saddle pulmonary embolism.

## Introduction

Since late 2019, the world is facing a rapidly expanding pandemic of a lower respiratory infection caused by severe acute respiratory syndrome coronavirus 2 (SARS-CoV-2) and causing a clinical syndrome entitled coronavirus disease 2019 (COVID-19). Clinical symptoms of the virus are vague and can mimic any other respiratory infection. However, what distinguishes this virus from others is its significant association with thromboembolic diseases as well as its high mortality rate. Recent findings revealed that patients with severe cases of COVID19 who are admitted to the intensive care unit with respiratory failure had a predominant hypercoagulable state leading to thromboembolism [1]. In fact, the infection causes dysfunction within the endothelium as well as an inflammatory state leading to increased thrombin formation and reduced fibrinolysis [2]. We present a case of a healthy patient who presented to the hospital with dyspnea, was found to be COVID-19 positive, and was diagnosed with an acute saddle pulmonary embolism.

## Case presentation

A 32-year-old male with no pertinent past medical history presented to the hospital with shortness of breath. He reports being tested positive for COVID-19 as an outpatient. At the time, he complained of some mild dyspnea and a non-productive cough, and kept himself quarantined for a few days until his dyspnea worsened which prompted him to come to the emergency department. He endorsed a single episode of hemoptysis, one teaspoon worth. Otherwise denied any fever, chills, nausea, vomiting, abdominal pain, and lower extremity tenderness or swelling. On examination, he was tachycardic with a heart rate of 130/minute, tachypneic with a respiratory rate of 30/minute, normotensive with a blood pressure of 128/86 mmHg, and his oxygen saturation was 92% on room air. He was tested with the COVID-19 polymerase chain reaction and was found to be positive.

**Figure 1:**
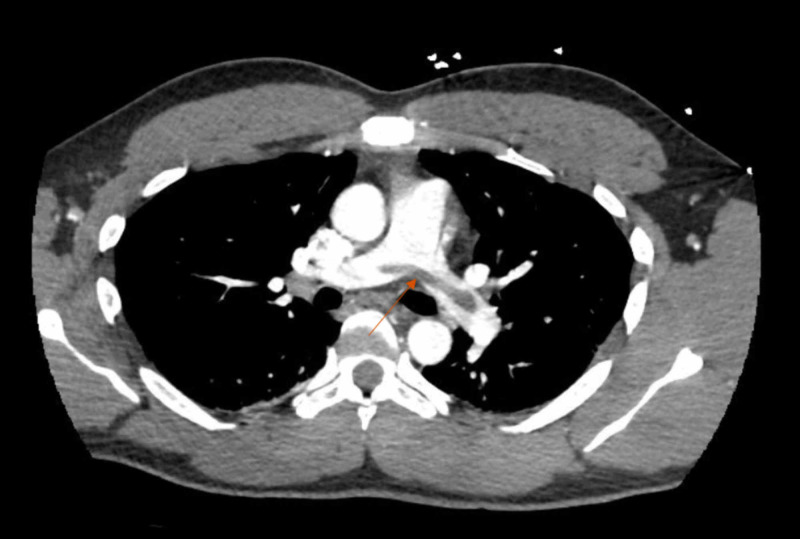
A CT angiography of the chest showing a large saddle pulmonary embolism (arrowhead)

Laboratory results were significant for creatinine 1.44 mg/dL (normal range: 0.70-1.20 mg/dL), procalcitonin 0.06 ng/mL (normal range: <0.10 ng/mL), ferritin 819 ng/mL (normal range: 30-400 ng/mL), pro-brain natriuretic peptide (proBNP) 38 pg/mL (normal range : <125 pg/mL), C-reactive protein 54.1 mg/L (normal range: <8.0 mg/L), and D-dimer >20 μg/mL (normal range: <0.50 μg/mL). A chest x-ray did not show any acute disease process. Electrocardiography (EKG) was performed and showed sinus tachycardia. A thoracic CT scan angiography (Figure 1) was performed showing large saddle embolism which extended into segmental branches in both lower lobes and the right middle lobe. There was also moderate peripheral non- enhancing consolidation seen at the left lung base posteriorly suggestive of a probable pulmonary infarct. He was diagnosed as having a submassive pulmonary embolism. The right ventricle (RV) was enlarged and was consistent with right-sided cardiac strain. A Doppler ultrasound of both lower extremities was negative for any deep vein thrombosis. The patient was started on a heparin drip and was admitted to the general medicine department. He was then shortly transferred to the intensive care unit for higher-level care and airway protection due to multiple episodes of hemoptysis and worsening tachypnea. 

**Figure 2:**
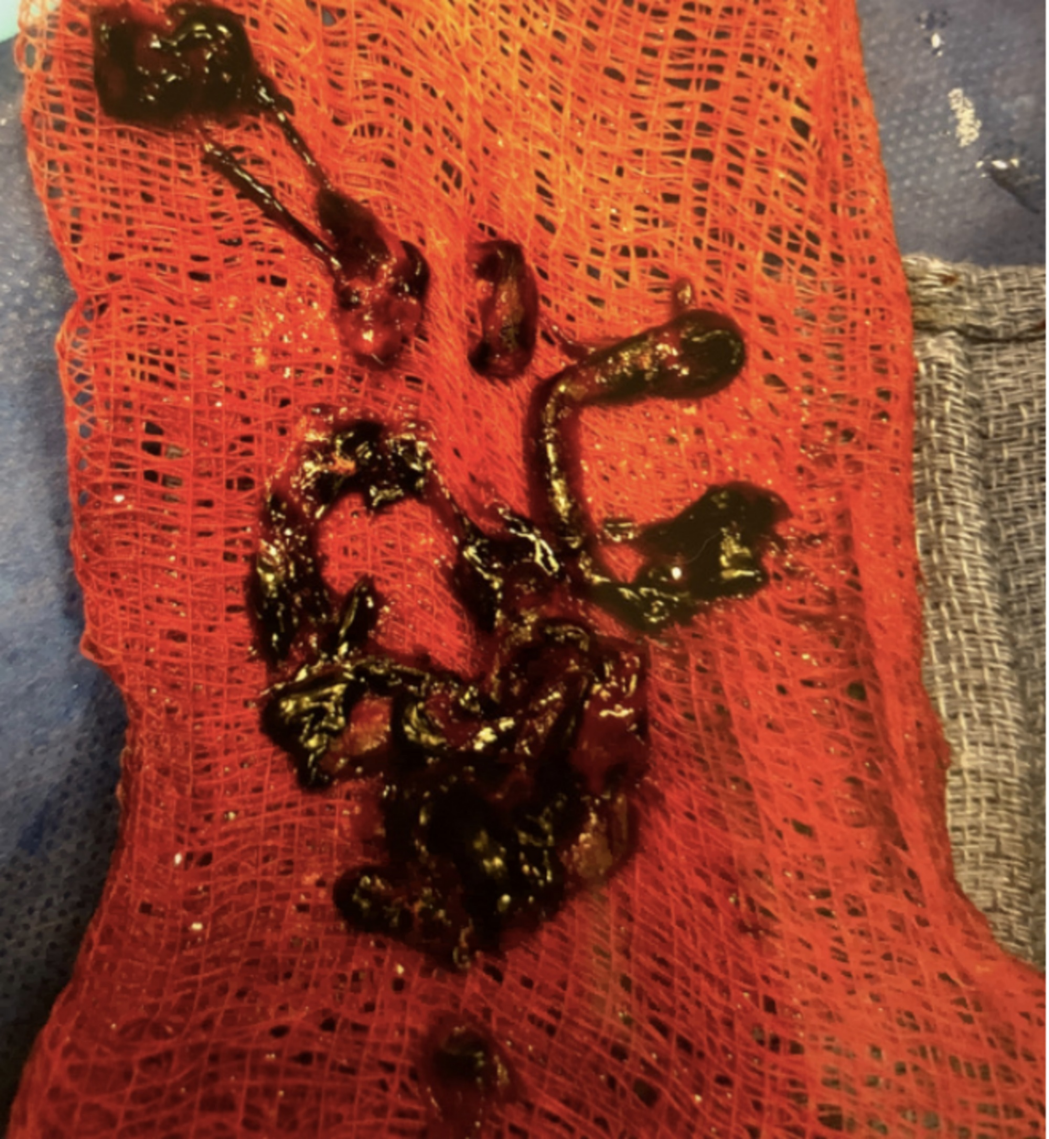
Saddle pulmonary embolism following mechanical embolectomy

Bedside echocardiogram performed by the cardiologist revealed a dilated RV consistent with the right heart strain. Vascular surgeons were consulted and decided to proceed with mechanical embolectomy since there was increasing evidence supporting catheter-directed therapy for submassive pulmonary embolism, which may have a lower risk of bleeding compared to systemic thrombolysis. A mechanical embolectomy was performed, and a large thrombus was removed (Figure 2). A subsequent pulmonary arteriogram demonstrated resolution of the filling defects. Postprocedure, the patient remained clinically stable. The patient was subsequently transitioned from a heparin drip to rivaroxaban 15 mg twice a day for 21 days followed by rivaroxaban 20 mg daily and was discharged with a three-month follow-up. 

## Discussion

The COVID-19 outbreak has been an exceptional universal public health challenge. As of mid-June 2020, there are about 7.55 million cases worldwide with a death toll reaching 423,000. A study conducted by Guan et al. that included 1,099 patients revealed that the most common symptoms of patients with COVID-19 were fever (88.7%) and cough (67.8), while diarrhea only occurring in 3.8% of the cases [3].

Pulmonary embolism and deep vein thrombosis have been reported in viral pneumonia caused by different viruses but not as frequently as in COVID 19 patients. One retrospective study performed by Bompard et al. reported that out of 137 patients who underwent CT pulmonary angiography, 50% of them were found to have acute pulmonary embolism [4]. It has been hypothesized that the COVID-19 induced thrombosis is partly due to the disease-specific hypercoagulable state and partly due to cytokine-mediated diffuse microvascular damage [5]. The risk of pulmonary embolism can be further increased with obesity, advanced age, and immobilization in hospitals. 

The incidence of acute pulmonary embolism in a patient with COVID-19 remains unknown [6]. However, the data available promote concerns about an increased incidence of thromboembolic diseases associated with the virus. Our patient was a healthy male who presented with no risk factors for thromboembolic disease and no symptoms of viral pneumonia but still developed a large saddle embolism.

## Conclusions

Given the large number of COVID-19 patients seeking medical care and requiring hospitalization, patients with known COVID-19 should receive a D-dimer test and if elevated on admission, CT pulmonary angiography should be considered since the incidence of thromboembolic diseases appears to be significantly higher in COVID-19. Pulmonary embolism is a life-threatening but potentially treatable disease that requires a high index of suspicion and benefits from early intervention.
